# Constitutional mosaicism for a *BRCA2* mutation as a cause of early-onset breast cancer

**DOI:** 10.1007/s10689-020-00186-1

**Published:** 2020-05-28

**Authors:** Pia Alhopuro, Reetta Vainionpää, Anna-Kaisa Anttonen, Kristiina Aittomäki, Heli Nevanlinna, Minna Pöyhönen

**Affiliations:** Department of Clinical Genetics and HUSLAB Laboratory of Genetics, Helsinki University Hospital, Helsinki, Finland

**Keywords:** *BRCA1*, *BRCA2*, Mosaicism, Breast cancer, De novo, Next-generation sequencing

## Abstract

Germline mutations in the *BRCA1* and *BRCA2* genes cause hereditary breast and ovarian cancer syndrome (HBOC). Mutations in these genes are usually inherited, and reports of *de novo BRCA1/2* mutations are rare. To date, only one patient with low-level *BRCA1* mutation mosaicism has been published. We report on a breast cancer patient with constitutional somatic mosaicism of a *BRCA2* mutation. *BRCA2* mutation c.9294C>G, p.(Tyr3098Ter) was detected in 20% of reads in DNA extracted from peripheral blood using next-generation sequencing (NGS). The *BRCA2* mutation was subsequently observed at similar levels in normal breast tissue, adipose tissue, normal right fallopian tube tissue and ovaries of the patient, suggesting that this mutation occurred early in embryonic development. This is the first case to report constitutional mosaicism for a *BRCA2* mutation and shows that *BRCA2* mosaicism can underlie early-onset breast cancer. NGS for *BRCA1/2* should be considered for patients whose tumors harbor a *BRCA1/2* mutation and for individuals suggestive of genetic predisposition but without a family history of HBO.

## Introduction

*BRCA1-* and *BRCA2-*associated hereditary breast and ovarian cancer syndrome (HBOC) is characterized by an increased susceptibility to breast and ovarian cancer, and to a lesser extent certain other cancers, especially in individuals with a *BRCA2* germline mutation. Pathogenic variants in these two genes are suggested to account for approximately 25% of inheritable breast cancers [1]. *BRCA1* and *BRCA2* are the most commonly tested genes in individuals presenting with early-onset breast cancer, triple-negative breast cancer, bilateral breast cancer and familial breast/ovarian cancer [2].

Mutations in *BRCA1/2* are inherited in an autosomal dominant manner, and very few cases of de novo mutations have been reported. The haplotypes of many recurring *BRCA1/2* mutations have a common ancestral origin and some are known to be hundreds of years old. Pathogenic *BRCA1/2* variants thus represent a mixture of rare private mutations, some of which may be recent, and more common mutations passed down through several generations [3]. To the best of our knowledge, no patients with *BRCA2* constitutional mosaicism have been described in the literature, and a single patient with low-level constitutional mosaicism of *BRCA1*-mutation has been published [4].

The development of next-generation sequencing (NGS) technologies has revealed a significant contribution of mosaic mutations to cancer predisposition in an increasing number of patients, such as in Li-Fraumeni syndrome and familial adenomatous polyposis [5, 6]. In other cancer predisposition syndromes, including Lynch syndrome and HBOC, de novo mutations and mosaicism appear to be less frequent [7]. While this phenomenon may be rare in HBOC, it is important to recognize it for proper clinical management and genetic counselling.

## Materials and methods

A 56-year-old female who had developed ductal carcinoma of the left breast at the age of 36 years was referred to the Department of Clinical Genetics at Helsinki University Hospital. Her DNA extracted from peripheral blood lymphocytes and tissue specimens was analyzed for a *BRCA1* and *BRCA2* mutations using Ion AmpliSeq BRCA1 and BRCA2 Panel and Ion Torrent semiconductor sequencing (Ion Proton system, Thermo Fisher Scientific, Carlsbad, CA, USA). MLPA analysis was performed in parallel to exclude large deletions and duplications, employing Salsa MLPA probe mixes according to the manufacturer’s protocols (MRC-Holland, the Netherlands). BRCA2 variant was validated by Sanger sequencing using the primers F: 5′-CTCCTGTTAGCAATGTGTGCG-3′ and R: 5′-CCAAAATGTGTGGTGATGCTG-3′. The study was performed in accordance with the Declaration of Helsinki and approved by the Ethical Review Board of Helsinki University Hospital. Informed consent was obtained from the patient.

## Results

Histological analysis of the patient’s tumor specimen had confirmed an invasive ductal carcinoma, G3 (papillary, 17 mm), with no lymph nodes affected (0/16). In immunohistochemistry the tumor was triple-negative with absent staining for estrogen receptor, progesterone receptor and HER2. Ki-67 and p53 stainings were positive. The patient had undergone radical mastectomy and axillary lymphadenectomy, and 20 years after the initial diagnosis, there had been no recurrence.

**Fig. 1:**
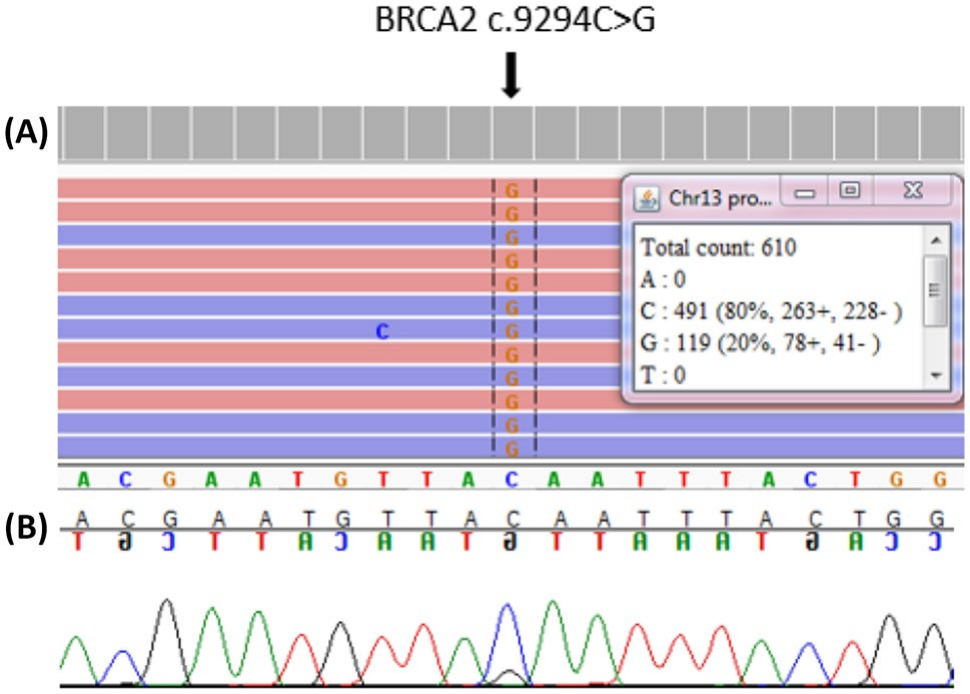
Sequencing analysis of the patient’s blood sample. **a** NGS reads and **b** Sanger sequencing confirmation of the NGS data showing the *BRCA2* mutation c.9294C>G in mosaic form

NGS analysis of patient’s peripheral blood DNA revealed a *BRCA2* (NM_000059.3) nonsense mutation c.9294C>G, p.(Tyr3098Ter) leading to a premature termination codon in 20% of reads. This variant is predicted to result in a truncated protein or mRNA subjected to nonsense-mediated decay. It has been observed in multiple individuals with breast and/or ovarian cancer (also denoted *BRCA2* 9522C>G in the literature) and has been classified pathogenic by ENIGMA-consortium in the ClinVar and BRCA Exchange databases [8]. MLPA analysis gave a normal result. To exclude technical errors, the mutation was subsequently re-analyzed in leucocyte DNA extracted from a second venipuncture, confirming the mutation in 20% of reads. In parallel with NGS, the sample was tested using Sanger sequencing, which revealed very weak signals representing the *BRCA2* c.9294C>G mutation (Fig. 1). Following the identification of the *BRCA2* mutation, the patient received genetic counselling. The putative mosaic nature of this mutation was discussed. Subsequently, the patient chose to undergo prophylactic mastectomy and salpingo-oophorectomy. In histological analysis the surgically removed tissues were cancer-free. DNA from five additional sites was thereafter extracted from fresh tissue and sequenced using NGS. These analyses revealed the *BRCA2* mutation c.9294C>G in 36% of reads derived from the right mammary gland tissue and in 25–29% of reads derived from the right fallopian tube, left and right ovaries and adipose tissue (Table 1). Breast tumor DNA was subsequently extracted from a paraffin-embedded tissue block, containing approximately 40% of tumor cells, and *BRCA2* c.9294C>G was detected in 57% of reads (Table 1).

Patient’s maternal aunt had deceased at age 80 years and had been diagnosed with breast cancer. In the first or second degree relatives, there were no additional malignancies. DNA extracted from peripheral blood of proband’s mother, aged 89 years, was tested for the *BRCA2* c.9294C>G mutation, with negative results. The proband’s father had died at age 80 of coronary heart disease. His DNA was not available for analysis. Examination of SNPs on the reads spanning the *BRCA2* c.9294C>G mutation were uninformative to determine the phase of the allele. Due to young age, genetic testing of patient’s two offspring was deferred.

## Discussion

In this patient constitutional mosaicism for a pathogenic *BRCA2* variant c.9294C>G was identified as a cause of genetic predisposition to HBOC. The mosaic nature of the mutation was confirmed in two independent leucocyte DNA samples using NGS. In parallel, the sample was analyzed using Sanger sequencing to exclude any technical errors (Fig. 1). Detection of *BRCA2* c.9294C>G mutation in several non-cancerous tissues of this individual excluded circulating tumor cells and clonal hematopoiesis as an origin. The mutation has most likely arisen de novo as an early postzygotic mutational event, as tissues originating from at least two germ layers were similarly involved (Table 1). Mesodermal derivatives include blood, ovary and adipose tissue, whereas e.g. mammary gland is of ectodermal origin. The tumor tissue was investigated to look for loss of heterozygosity (LOH) or a second hit. NGS analysis of paraffin-embedded tissue block, containing approximately 40% of malignant cells, revealed no additional *BRCA2*-mutations. The variant allele frequency of *BRCA2* c.9294C>G was 57% in tumor tissue suggesting possible somatic copy number change at this locus (Table 1). Since paternal sample was not available for analysis, revertant mosaicism, a spontaneous correction of a paternally inherited pathogenic mutation leading to somatic mosaicism, could not be excluded. Revertant mosaicism is rare overall but a well-described phenomenon particularly in hematological conditions and skin diseases [9]. It has never been observed in HBOC or in phenocopies of *BRCA1/2* families [10].

The prevalence of de novo and mosaic mutations in HBOC seems to be low. However, this phenomenon may have been slightly underestimated, as the family history of the proband is typically taken into account as a selection criterion for genetic testing. Furthermore, some cases may have been missed prior to the use of NGS due to the limitations of Sanger sequencing to identify low-level mosaicism. In our patient, the age of onset and triple negativity were highly suggestive of genetic predisposition. To date, only a dozen of patients with *de novo BRCA1/2* mutations have been reported [4, 10]. Interestingly, the *de novo BRCA1/2* mutations described in the literature have typically been identified in patients with early-onset cancer, possibly reflecting selection bias. Detecting de novo mosaic mutations is important in terms of genetic counselling. While siblings and parents of the proband will not be affected, the risk of transmitting the mutation to offspring depends on the level of mosaicism in patient’s germ cells, and may be different from the 50% chance in individuals with germline mutation. Mosaicism can contribute to the predicted phenotype, however, correlation between disease severity and mosaicism level in leucocytes is inconclusive.

NGS technology has enabled high-fold coverage of sequenced fragments and quantification of variant allele frequencies (VAFs). Low VAF alone, however, is not sufficient to establish somatic mosaicism in a patient. Additional tissue material needs to be tested to distinguish between the different etiologies. Considering the clinical implications, clinical laboratories should establish protocols to ensure detection of mosaic mutations and policies for verification of the result from additional material, such as buccal swabs, saliva or fibroblasts.

**Table 1:** Tissue distribution of *BRCA2* mutation c.9294C>G

Tissue type	Read depth	VAF (%)
Peripheral blood (draw 1)	2566	20
Peripheral blood (draw 2)	610	20
Mammary gland (right)	4732	36
Fallopian tube (right)	6893	29
Right ovary	3332	21
Left ovary	6315	25
Adipose tissue	6783	26
Breast cancer	308	57

Although mosaicism for *BRCA1/2* mutations seems to be rare, this and previous work demonstrates that low-level mosaic mutations can contribute to the etiology of breast cancer susceptibility [4]. This notion may have important implications in selected patients, and calls for additional attention to define the extent of this phenomenon. This study highlights the power of deep sequencing in detecting somatic mosaic mutations in various tissues and demonstrates the need to consider NGS in genetic testing of individuals suspected of carrying a germline cancer predisposition. Especially in germline testing of patients, who have a verified somatic pathogenic *BRCA1/2* mutation in tumor tissue, or with no family history of HBOC but personal history suggestive of genetic predisposition, methods capable of identifying low-level mosaicism should be used.
